# Rising clustering of metabolic risk factors and behavioral-metabolic profiles in Viet Nam, 2015–2021: repeated cross-sectional surveys

**DOI:** 10.3389/ijph.2026.1609281

**Published:** 2026-06-19

**Authors:** Duy Quang Pham, Thi Tu Quyen Bui, Hung Minh Nguyen, Minh Van Hoang

**Affiliations:** 1 Faculty of Science, University of British Columbia, Vancouver, BC, Canada; 2 Department of Biostatistics, Hanoi University of Public Health, Hanoi, Vietnam; 3 Vietnam National Heart Institute, Bach Mai Hospital, Hanoi, Vietnam; 4 Department of Health Ecomic, Hanoi University of Public Health, Hanoi, Vietnam

**Keywords:** latent class analysis, metabolic risk factors, noncommunicable diseases, risk factor clustering, STEPS survey

## Abstract

**Objectives:**

Evidence on the burden and clustering of metabolic non-communicable disease risks and on data-driven risk profiles remains limited. This study assessed trends in the clustering of metabolic risk factors in Viet Nam from 2015 to 2021 and identified behavioral–metabolic profiles in 2021.

**Methods:**

A repeated cross-sectional design was used, using national STEPS data from 2015 (n = 3,074) and 2021 (n = 3,712), both of which included participants who completed all survey components. Behavioral risk factors (smoking, alcohol use, physical inactivity, and low fruit and vegetable intake) and metabolic risk factors (elevated blood pressure, fasting glucose, body mass index (BMI), and total cholesterol) were defined using Asian-calibrated cutoffs. Clustering was defined as the co-occurrence of two or more metabolic risk factors within an individual. Weighted analyses estimated prevalence and clustering, and latent class analysis (LCA) identified behavioral–metabolic profiles.

**Results:**

All metabolic risks increased between 2015 and 2021, most sharply for raised fasting glucose (12.0% → 37.7%). This increase was a major contributor to intensified clustering, with the prevalence of ≥2 risk factors increasing from 27.6% to 46.6% and ≥3 risk factors increasing from 8.7% to 20.1%. LCA revealed three profiles: “Health-Conscious” (38.1%, low behavioral and metabolic risks), “Metabolic Risk-Aware” (34.6%, low behavioral but high metabolic risks, linked to older age), and “High Behavioral Risk with Moderate Metabolic Comorbidity” (27.3%, characterized by high smoking and alcohol use together with moderate metabolic abnormalities, concentrated among socioeconomically disadvantaged men).

**Conclusion:**

Metabolic risks in Viet Nam increased markedly between 2015 and 2021, with elevated glucose levels playing an important role in multi-risk clustering. These distinct profiles underscore the need for integrated, multi-risk screening and management in primary care, especially targeting older adults and socioeconomically disadvantaged men.

## Introduction

Noncommunicable diseases (NCDs) are the leading cause of morbidity and mortality worldwide, responsible for about 71% of all deaths, with low- and middle-income countries (LMICs) bearing about 85% of premature NCD deaths [1]. Viet Nam has undergone a rapid epidemiological transition: about 72% of deaths and about 66% of total disease burden are attributable to NCDs, notably cardiovascular diseases, cancer, diabetes, hypertension, and chronic obstructive pulmonary disease (COPD) [2]. Modifiable upstream drivers include tobacco and alcohol use, physical inactivity, and unhealthy diet; downstream biological abnormalities, elevated blood pressure, elevated fasting blood glucose, raised cholesterol, and overweight/obesity, mediate much of the burden. These are the targets of the World Health Organization’s “best buys” for NCD prevention and control [3].

Previous studies show that NCD risks increasingly co-occur within individuals [4, 5]. This risk-factor clustering, in which two or more risks co-occur in the same person, has been linked to earlier onset and greater severity of NCDs [6, 7]. Planning, diagnostics/consumables, and human resources, therefore, require data not only on single risk factors but also on how metabolic risks cluster within individuals and how that clustering changes over time [8].

While WHO STEPS surveys provide standardized surveillance of behavioral and metabolic risk factors, previous national analyses in Viet Nam [9, 10] either describe each factor in isolation or fit predictive models of metabolic outcomes on behaviors. Some studies in other countries [4, 11, 12] have reported clustering risk, but it is often operationalized as a simple sum of risks rather than using model-based methods to detect hidden population profiles. Such approaches are limited because (i) without joint data on risk factors, monitoring NCDs and planning treatment are difficult; and (ii) in cross-sectional data, regression of “metabolic outcome on behavior” may be biased by behavior change after symptom onset or diagnosis (e.g., persons with hypertension reducing alcohol or salt), obscuring the behavioral-metabolic patterns relevant for prevention and chronic care design [13]. Furthermore, previous research [9, 10] often employed conventional clinical cut-offs (BP ≥ 140/90 mmHg, fasting blood glucose ≥7 mmol/L, Body Mass Index (BMI) ≥ 25 kg/m^2^, or total cholesterol ≥6.2 mmol/L), which may underestimate risk in Asian populations [13, 14].

This study aims to address key gaps in understanding NCD risk in Viet Nam by (i) providing comprehensive estimates of the prevalence of both individual and clustered metabolic abnormalities over time (2015–2021) using Asian-calibrated risk thresholds and (ii) applying Latent Class Analysis (LCA) to identify data-driven behavioral-metabolic profiles and the population groups most likely to belong to each profile. These findings support more efficient resource allocation, medicines, diagnostics, and staffing, and inform integrated, multi-risk-factor strategies aligned with WHO priorities [15] and national priorities. As Viet Nam’s STEPS data use internationally standardized methods, this study can provide useful information for other LMICs navigating similar epidemiologic and health system transitions.

## Methods

### Study design and data source

This analysis employed a repeated cross-sectional study, using national data from the 2015 and 2021 STEPS surveys conducted in Viet Nam. Both surveys followed the standardized WHO STEPwise approach to NCD risk factor surveillance [16] and included individuals aged 15 years and older. A two-stage stratified cluster sampling design was applied, using Enumeration Areas as the primary sampling units, to ensure national representativeness across all 63 provinces and cities. For STEPS 2 and 3, anthropometric and biochemical indicators were collected only from participants aged 18 years and older. Accordingly, this study focused on respondents aged 18 and above who completed all three components of the survey (interview, physical measurements, and blood test), yielding analytic samples of 3,074 in 2015 and 3,712 in 2021. For latent class analysis (LCA), we restricted the sample to STEPS 2021 participants with complete data on all behavioral risk factors and required blood tests, yielding a final analytic sample of n = 3,306. LCA was applied to the 2021 dataset only because it provides the most recent cross-sectional snapshot of population risk profiles and ensures consistency in measurement across all indicators.

### Study measurement

Four NCD behavioral risk factors were examined: current smoking, current drinking, insufficient physical activity, and low consumption of fruits and vegetables. Current smoking was defined based on the question “Do you currently smoke tobacco on a daily basis, less than daily, or not at all?”; participants who reported daily or less-than-daily use were classified as current smokers. Current drinking was defined as having consumed at least one standard alcoholic drink within the past 30 days. Physical activity was measured according to the WHO recommendation of at least 600 MET-minutes per week across work, transport, and leisure domains; participants with a total score below this threshold were considered not to meet the recommendation. Finally, fruit and vegetable intake was assessed as the average number of servings per day; fewer than 5 servings per day were classified as insufficient. To standardize data collection, interviewers used visual showcards (for tobacco, alcohol, fruits, vegetables, and physical activity) to help participants report quantities and frequencies in standard units. Data were collected using electronic data capture systems in both survey rounds; the 2021 survey used a REDCap mobile application with integrated questionnaires and showcards, while the 2015 survey used an earlier electronic platform.

Four metabolic abnormalities were assessed: hypertension, elevated fasting blood glucose, overweight/obesity, and elevated total cholesterol. Hypertension was defined as systolic blood pressure ≥130 mmHg, diastolic blood pressure ≥85 mmHg, or current use of antihypertensive medication [17]. Elevated fasting blood glucose (indicative of diabetes or prediabetes) was defined as ≥5.6 mmol/L or current use of diabetes medication. Overweight/obesity (BMI ≥23 kg/m^2^) was classified according to cutoffs commonly used for Asian populations. Elevated total cholesterol was defined as a concentration of ≥5.2 mmol/L [13, 14]. Physical measurements were obtained using standardized WHO-recommended equipment, including digital blood pressure monitors (BOSO), electronic weighing scales (Seca), and stadiometers and measuring tapes for anthropometry. Biochemical measurements, including fasting blood glucose and total cholesterol, were conducted using point-of-care devices (CardioChek Plus) following standardized STEPS protocols, with consistent procedures applied across both survey rounds.

Demographic characteristics were assessed across several domains. Age was categorized into five groups: 18–24 years, 25–34 years, 35–44 years, 45–54 years, and ≥55 years. Education level was classified as primary school or less, secondary school, high school, university/college, or no information. Marital status was categorized into three groups: not married, married, and divorced/widowed. Occupation referred to the main activity over the past 12 months and included working for a government or non-government agency/organization, being self-employed, being a student, being a homemaker, being retired, or being in another category. Sex was recorded as male or female, place of residence as urban or rural, and ethnicity as Kinh or other. Socioeconomic status was measured using the household wealth index, constructed through principal component analysis of household assets and housing characteristics. This index is standardized in many large household surveys in low- and middle-income countries and has been validated in Viet Nam [18]. The index was divided into quintiles, ranging from the poorest (Q1) to the richest (Q5).

### Statistical analysis

We used the svy procedures in Stata 18 to estimate the survey-weighted prevalence and 95% confidence intervals (CIs) for all NCD risk factors and the clustering of metabolic abnormalities for both 2015 and 2021. Survey weights were applied in descriptive prevalence analyses to account for the complex sampling design. For clustering patterns, we present: (i) the proportion of individuals with zero, one, two, three, or four metabolic abnormalities and (ii) the most common two- and three-factor combinations.

To identify latent risk profiles, we conducted a latent class analysis (LCA) using 2021 data, incorporating all 8 binary indicators (4 behavioral and 4 metabolic). These indicators were selected *a priori* based on their established role as core behavioral and metabolic risk factors within the WHO STEPS framework and their availability across survey components [16]. The LCA models were estimated without directly incorporating survey weights or complex survey design variables because of methodological limitations in latent class estimation with gsem and lclass () in Stata. We evaluated candidate models with 2–7 classes using model fit criteria, including the Bayesian Information Criterion (BIC), Akaike Information Criterion (AIC), sample-size adjusted BIC, entropy, and clinical interpretability. Model fit statistics showed that the three-class model had the lowest Bayesian Information Criterion (BIC), indicating the best balance between model fit and parsimony. Although AIC continued to decrease with additional classes, improvements beyond three classes were modest. Entropy values were similar across models, suggesting no clear gain in classification quality with more complex solutions. Therefore, the three-class model was selected based on BIC, parsimony, interpretability, and conceptual coherence (see Supplementary Material 1). We report class-specific item-response probabilities and estimated population proportions (with 95% CIs). Classes were labeled based on dominant response patterns as follows: “Health-Conscious Group” (Class 1), “Metabolic-Risk Aware” (Class 2), and “High Behavioral Risk with Moderate Metabolic Comorbidity” (Class 3).

Participants were assigned to their most likely latent class using modal posterior probabilities. We then used multinomial logistic regression to examine associations between class membership and sociodemographic characteristics, treating Class 1 as the reference category. Covariates included age group, sex, education level, ethnicity, urban/rural residence, and household socioeconomic status (measured in quintiles), selected *a priori* based on epidemiological relevance and data availability. Results are presented as Odds Ratios (ORs) with 95% CIs and p-values, using a significance threshold of *p* = 0.05. A sensitivity analysis restricted to individuals with high classification certainty (posterior probability >0.80) yielded similar findings in both direction and magnitude.

### Role of the funding source

There was no funding source for this study.

### Ethical consideration

This study analyzed de-identified secondary data from the 2015 and 2021 WHO STEPwise (STEPS) surveys in Viet Nam. The national STEPS surveys received ethics approval from the Ministry of Health and the Hanoi University of Public Health, and written informed consent was obtained from all participants before data collection. Since the datasets were fully de-identified, this secondary analysis did not require additional approval from the institutional review board.

## Results

### Characteristics of the study sample

The analytic sample comprised 3,074 participants in 2015 and 3,712 in 2021, aged 18 years and older. In both survey years, the largest age groups were 35–44 and 45–54 years. Most participants were married and identified as members of the Kinh ethnic group. Educational attainment ranged from primary to tertiary, with a substantial proportion having completed high school or higher. Self-employment was the most common occupation. Both men and women were included, with a near-equal sex distribution in 2021. Urban and rural residents were well-represented across the survey years. Household socioeconomic status was relatively evenly distributed across quintiles in both survey years. However, the proportion in the middle quintile (Q3) increased from 15.7% in 2015 to 21.1% in 2021, while the proportions in the lower quintiles declined modestly. Detailed sociodemographic characteristics are presented in Table 1.

**TABLE 1:** Characteristics of survey sample in WHO STEPS, Viet Nam, 2015 and 2021.

Factors	Year 2015 (N = 3,074), n (%)	Year 2021 (N = 3,712), n (%)
Age group		
18–24	259 (8.4)	196 (5.3)
25–34	583 (19.0)	528 (14.2)
35–44	767 (25.0)	851 (22.9)
45–54	724 (23.6)	898 (24.2)
over 55	741 (24.1)	1,239 (33.4)
Education group		
Primary school or less	565 (18.4)	1,479 (39.8)
Secondary school	683 (22.2)	952 (25.6)
High school	856 (27.8)	679 (18.3)
University/college	954 (31.0)	602 (16.2)
No information	16 (0.5)	0 (0.0)
Marital status		
Not married	302 (9.8)	280 (7.5)
Married	2,453 (79.8)	2,989 (80.5)
Divorced/Widower	319 (10.4)	443 (11.9)
Occupation during the last 12 months		
Working for a government agency/organization	276 (9.0)	274 (7.4)
Working for a non-government agency/ organization	283 (9.2)	166 (4.5)
Self-employed	1914 (62.3)	2,452 (66.1)
Student	49 (1.6)	53 (1.4)
Homemaker	257 (8.4)	304 (8.2)
Retired	158 (5.1)	295 (7.9)
Other	137 (4.5)	168 (4.6)
Sex		
Men	1,317 (42.8)	1817 (48.9)
Women	1757 (57.2)	1895 (51.1)
Urban		
Urban	1,377 (44.8)	1760 (47.4)
Rural	1,697 (55.2)	1952 (52.6)
Ethnicity group		
Kinh	2,514 (81.8)	3,054 (82.3)
Others	560 (18.2)	658 (17.7)
Household SES		
Q1	671 (21.8)	737 (19.9)
Q2	754 (24.5)	844 (22.7)
Q3	484 (15.7)	783 (21.1)
Q4	632 (20.6)	664 (17.9)
Q5	533 (17.3)	684 (18.4)

### Prevalence of NCD behavioral and metabolic risk factors in 2015 and 2021

Table 2 presents the prevalence of risk factors in 2015 and 2021 among individuals who completed all three rounds of the STEPS survey. For behavioral risk factors, current smoking showed a slight decline, from 25.54% (95% CI: 23.58–27.50) to 22.56% (20.62–24.49), with overlapping confidence intervals suggesting no clear evidence of change; current drinking declined from 44.17% to 38.35%; low fruit and vegetable intake also decreased from 27.47% to 22.51%; while insufficient physical activity remained high with little change (56.87% in 2015% vs. 59.47% in 2021).

**TABLE 2:** Survey-weighted prevalence of noncommunicable disease risk factors, WHO STEPS, Viet Nam, 2015 and 2021^a^

Indicator (cut-off/definition)	Year 2015 (n = 3,074) Prevalence (95% Confidence interval)	Year 2021 (n = 3,712) Prevalence (95% Confidence interval)
Current smoking	25.54 (23.58, 27.50)	22.56 (20.62, 24.49)
Current drinking	44.17 (42.00, 46.42)	38.35 (36.04, 40.64)
Insufficient physical activity	56.87 (54.64, 59.11)	59.47 (57.13, 61.82)
Low fruit and vegetable intake	27.47 (25.42, 29.51)	22.51 (20.53, 24.49)
Hypertension	30.31 (28.35, 32.27)	41.13 (38.86, 43.40)
Elevated fasting blood glucose	11.99 (10.63, 13.36)	37.67 (35.25, 40.09)
Overweight/Obesity	33.87 (31.80, 35.94)	38.68 (36.37, 41.00)
Elevated total cholesterol	23.54 (21.73, 25.35)	35.26 (32.98, 37.53)

a All estimates are restricted to participants who completed all three STEPS (questionnaire, physical measurements, and biochemical tests). Therefore, behavioral risk-factor prevalences may differ from those in the official national STEPS reports, which are based on the full Step 1 sample.

All four metabolic indicators showed increasing trends over time: hypertension rose from 30.31% (28.35–32.27) to 41.13% (38.86–43.40); elevated fasting blood glucose increased sharply from 11.99% (10.63–13.36) to 37.67% (35.25–40.09), marking the largest absolute change; overweight/obesity (BMI ≥23 kg/m^2^) increased from 33.87% (31.80–35.94) to 38.68% (36.37–41.00); and elevated total cholesterol increased from 23.54% (21.73–25.35) to 35.26% (32.98–37.53).

### Clustering of NCD metabolic risk factors

Table 3 presents the clustering of metabolic risk factors across four abnormalities: overweight/obesity, hypertension, elevated fasting blood glucose, and elevated total cholesterol. The proportion of adults with no abnormality declined substantially from 40.2% (95% CI: 37.9–42.5) in 2015 to 23.3% (21.1–25.7) in 2021. Conversely, the prevalence of individuals with two or more metabolic abnormalities increased markedly from 27.6% to 46.6%. The share with exactly two abnormalities rose from 18.9% to 26.5%, while those with three or more abnormalities more than doubled, from 8.7% (three: 6.9%; four: 1.8%) to 20.1% (three: 15.7%; four: 4.4%).

**TABLE 3:** Survey-weighted prevalence of metabolic risk-factor clusters, WHO STEPS, Viet Nam, 2015 and 2021.

Risk factors	Year 2015 (n = 3,074) Prevalence (95% Confidence interval)	Year 2021 (n = 3,712) Prevalence (95% Confidence interval)	# risk factors
None	40.2% (37.9–42.5)	23.3% (21.1–25.7)	0
Overweight/obesity	11.9% (10.3–13.7)	7.0% (5.8–8.3)	1
Hypertension	9.9% (8.6–11.3)	9.3% (8.1–10.6)	1
Elevated total cholesterol	7.7% (6.5–9.1)	7.6% (6.3–9.2)	1
Elevated fasting glucose	2.7% (2.1–3.6)	6.2% (4.9–7.8)	1
Hypertension + overweight/obesity	7.8% (6.7–9.1)	5.9% (5.0–7.1)	2
Overweight/obesity + elevated total cholesterol	4.2% (3.4–5.2)	4.4% (3.3–5.9)	2
Hypertension + elevated total cholesterol	3.2% (2.5–3.9)	4.7% (3.8–5.7)	2
Hypertension + elevated fasting glucose	1.4% (1.0–1.9)	4.4% (3.4–5.5)	2
Elevated fasting glucose + overweight/obesity	1.2% (0.8–1.7)	3.8% (3.0–4.9)	2
Elevated fasting glucose + elevated total cholesterol	1.1% (0.8–1.6)	3.3% (2.5–4.3)	2
Hypertension + overweight/obesity + elevated total cholesterol	4.4% (3.6–5.4)	4.5% (3.7–5.5)	3
Hypertension + elevated fasting glucose + overweight/obesity	1.4% (1.0–1.9)	4.9% (4.1–5.7)	3
Elevated fasting glucose + overweight/obesity + elevated total cholesterol	0.6% (0.4–1.0)	3.3% (2.4–4.6)	3
Hypertension + elevated fasting glucose + elevated total cholesterol	0.5% (0.3–0.8)	3.0% (2.4–3.8)	3
Hypertension + elevated fasting glucose + overweight/obesity + elevated total cholesterol	1.8% (1.3–2.3)	4.4% (3.6–5.4)	4

Combinations involving elevated fasting blood glucose increased over time: the prevalence of hypertension and elevated fasting blood glucose rose from 1.4% to 4.4%, overweight/obesity and elevated fasting blood glucose from 1.2% to 3.8%, and elevated fasting blood glucose and elevated total cholesterol from 1.1% to 3.3%. Triple clusters including glucose (e.g., hypertension, elevated fasting blood glucose, and overweight/obesity) increased from 1.4% to 4.9%, and the share with all four abnormalities more than doubled, from 1.8% to 4.4%.

### Latent class analysis of behavioral and metabolic risk patterns in 2021

In 2021, LCA identified three distinct classes of behavioral and metabolic risk among participants. Class 1, “Health-Conscious” (38.1%), showed low probabilities across all risk factors, including smoking, alcohol use, physical inactivity, low fruit and vegetable intake, and metabolic indicators. Class 2, “Metabolic Risk-Aware” (34.6%), had low behavioral risk but higher levels of metabolic abnormalities, including overweight/obesity, hypertension, elevated fasting blood glucose, and elevated total cholesterol. Class 3, “High Behavioral Risk with Moderate Metabolic Comorbidity” (27.3%), was characterized by high probabilities of smoking and alcohol use together with moderately elevated metabolic indicators. Class-specific item-response probabilities are presented in Table 4.

**TABLE 4:** Latent class analysis of lifestyle and metabolic risk patterns, WHO STEPS, Viet Nam, 2021^a^.

Variable	Health-conscious (%)	Metabolic risk-aware (%)	High behavioral risk with moderate metabolic comorbidity (%)
Class proportion (95% CI)	38.1 (31.2–45.4)	34.6 (27.7–42.1)	27.3 (22.8–32.5)
Current smoking	5.9%	5.3%	**70.5%** ^†^
Current alcohol drinking	18.2%	29.3%	**80.5%** ^†^
Low physical activity	24.8%	24.8%	12.7%
Low fruit/vegetable intake	**54.0%** ^†^	55.2%	**68.1%** ^†^
Hypertension	21.7%	**71.9%** ^†^	**60.8%** ^†^
Elevated fasting blood glucose	20.9%	**61.1%** ^†^	47.5%
Overweight/obesity	22.6%	**69.2%** ^†^	32.3%
Elevated total cholesterol	29.1%	**58.7%** ^†^	30.8%

a Values represent item-response probabilities (%) for each behavioral and metabolic risk factor within each latent class. Class proportions are presented as weighted estimates with 95% confidence intervals. Definitions of metabolic indicators follow Asian-specific thresholds described in the Methods.

^†^Bold values indicate the highest item-response probability for each risk factor across the three latent classes and were used to aid interpretation and labeling of the latent class profiles.

### Correlates of latent class membership

Older age was strongly associated with membership in both higher-risk classes. Compared with adults aged 18–24 years, individuals aged ≥55 years had substantially higher odds of membership in the “Metabolic Risk-Aware” class (OR: 25.97, 95% CI: 13.70–50.33, p < 0.001) and the “High Behavioral Risk with Moderate Metabolic Comorbidity” class (OR: 8.08, 95% CI: 4.90–13.72, p < 0.001).

Higher levels of education were associated with lower odds of membership in the higher-risk classes, particularly the “High Behavioral Risk with Moderate Metabolic Comorbidity” class. Individuals with university or college education had 57% lower odds of membership in this class (OR: 0.43, 95% CI: 0.30–0.63, p < 0.001) compared to those with primary education or less.

Women had significantly lower odds than men of membership in either higher-risk class, most notably for the “High Behavioral Risk with Moderate Metabolic Comorbidity class”, where the OR was 0.01 (95% CI: 0.01–0.02, p < 0.001).

Rural residence was associated with 30% lower odds of membership in the “Metabolic-Risk Aware” class (OR: 0.70, 95% CI: 0.58–0.84, p < 0.001), but no significant association was observed for the “High Behavioral Risk with Moderate Metabolic Comorbidity” class (p = 0.574).

Regarding household socioeconomic status, individuals in the richest quintile (Q5) had lower odds of membership in the “High Behavioral Risk with Moderate Metabolic Comorbidity” class than those in the poorest quintile (OR: 0.59, 95% CI: 0.39–0.90, p = 0.014). No significant socioeconomic gradient was observed for the “Metabolic-Risk Aware” group.

A sensitivity analysis restricted to individuals with high class assignment certainty (posterior probability >0.80) yielded similar results. While point estimates were modestly attenuated, particularly for age and SES, the overall direction and interpretation of the main associations (age, education, sex, and rurality) remained unchanged. Detailed multinomial regression results are presented in Table 5.

**TABLE 5:** Multinomial logistic regression of latent class membership comparing the two higher-risk groups with the health-conscious group, WHO STEPS, Viet Nam, 2021.

Variable	Category	Class 2 - metabolically risk-aware OR (95% CI), *p*	Class 3 - high behavioral risk with moderate metabolic comorbidity OR (95% CI), *p*
Age group (ref: 18–24)	25–34	4.28 (2.19–8.39) *p* < 0.001	2.25 (1.33–3.86) *p* = 0.003
	35–44	6.71 (3.52–12.99) *p* < 0.001	3.57 (2.17–6.02) *p* < 0.001
	45–54	13.54 (7.11–26.24) *p* < 0.001	5.82 (3.50–9.82) *p* < 0.001
	≥55	25.97 (13.70–50.33) *p* < 0.001	8.08 (4.90–13.72) *p* < 0.001
Education (ref: Primary or less)	Secondary school	0.87 (0.69–1.08) *p* = 0.211	0.62 (0.47–0.83) *p* = 0.001
	High school	0.70 (0.54–0.92) *p* = 0.009	0.60 (0.44–0.84) *p* = 0.002
	University/college	0.70 (0.53–0.94) *p* = 0.014	0.43 (0.30–0.63) *p* < 0.001
Ethnicity (ref: Kinh)	Other ethnicity	0.98 (0.76–1.25) *p* = 0.906	1.18 (0.90–1.64) *p* = 0.308
Sex (ref: Men)	Women	0.42 (0.34–0.50) *p* < 0.001	0.01 (0.01–0.02) *p* < 0.001
Residence (ref: Urban)	Rural	0.70 (0.58–0.84) *p* < 0.001	0.93 (0.73–1.17) *p* = 0.574
Household SES (ref: Q1 - poorest)	Q2	1.07 (0.79–1.44) *p* = 0.661	0.85 (0.65–1.23) *p* = 0.364
	Q3	1.12 (0.83–1.52) *p* = 0.469	0.64 (0.44–0.92) *p* = 0.017
	Q4	1.19 (0.86–1.65) *p* = 0.276	0.87 (0.58–1.31) *p* = 0.494
	Q5 (richest)	1.00 (0.71–1.43) *p* = 0.993	0.59 (0.39–0.90) *p* = 0.014

## Discussion

This study showed that the clustering of metabolic risk factors has intensified significantly over time. The proportion of adults with two or more metabolic abnormalities increased from 27.6% in 2015 to 46.6% in 2021, and those with three or more nearly tripled (from 8.7% to 20.1%). This suggests a shift from isolated risks toward increasingly complex multimorbidity profiles, indicating a rising burden of metabolic syndrome-like patterns in the population. Clusters involving elevated blood glucose, such as combinations with overweight/obesity or hypertension, showed the most pronounced increases, consistent with global concerns over the expanding burden of diabetes and prediabetes in LMICs [10, 19, 20]. Although both survey rounds followed standardized WHO STEPS protocols with consistent measurement procedures, the magnitude of increase in elevated fasting blood glucose should be interpreted with caution, as differences in measurement conditions (e.g., fasting status, sample handling, or field procedures) may have contributed to the observed change. The prevalence of NCD metabolic risk factors reported in this study is higher than in previous analyses of Viet Nam’s STEPS data [5, 9, 10]. This difference arises from our use of Asian-calibrated thresholds (SBP ≥130 mmHg or DBP ≥85 mmHg or antihypertensive medication; fasting glucose ≥5.6 mmol/L or diabetes medication; BMI ≥23 kg/m^2^), whereas earlier estimates applied conventional clinical thresholds (SBP ≥140 mmHg or DBP ≥90 mmHg; fasting glucose ≥6.1–7.0 mmol/L; BMI ≥25 kg/m^2^; total cholesterol ≥6.2 mmol/L). By applying thresholds more appropriate for Asian populations, our findings likely provide a more sensitive estimate of the population at risk.

More importantly, LCA identified three distinct behavioral-metabolic profiles in the adult population (18–69 years) in 2021. Class 1, “Health-Conscious” (38.1%), showed low probabilities across all behavioral and metabolic risks, as indicated by item-response probabilities (Table 4), representing a comparatively low-risk segment. Class 2, “Metabolic Risk-Aware” (34.6%), exhibited minimal behavioral risk but elevated metabolic markers (BMI, blood pressure, glucose, cholesterol). This class was markedly age-skewed: compared with adults aged 18–24 years old, those ≥55 had about 26 times higher odds of membership, and rural residents were less likely to belong. The profile is consistent with patterns observed in older adults or individuals with existing metabolic conditions, which may reflect behavior modification following diagnosis, rather than preventive health awareness alone, an adaptation commonly reported in previous studies, including Viet Nam [21–23]. Class 3, “High Behavioral Risk with Moderate Metabolic Comorbidity” (27.3%), was characterized by high smoking and alcohol use together with moderate metabolic abnormalities, consistent with the co-occurrence of behavioral and metabolic risk factors observed in prior studies [9, 24].

Correlates reinforce this interpretation: men had substantially higher odds of membership than women, higher education was associated with lower odds of membership in higher-risk classes, and the richest quintile had lower odds of membership design than the poorest, mirroring male-skewed clustering of smoking and alcohol use and socioeconomic gradients documented in regional literature [25, 26]. Together, these classes provide actionable segmentation: maintain and prevent relapse in Class 1 “Health-Conscious”; intensify secondary prevention and metabolic control for Class 2 “Metabolic Risk Aware”; and prioritize primary prevention and behavior change (especially targeting men with lower education and lower SES) in Class 3, “High Behavioral Risk with Moderate Metabolic Comorbidity”. Comparison with other LMICs highlights both commonalities and differences. A latent class analysis in Zambia also identified three NCD risk groups (low, intermediate, and high), which broadly paralleled our profiles. Their high-risk group resembled our “Metabolic Risk-Aware” class in terms of metabolic burden, but unlike in Viet Nam, Zambian participants had not modified behaviors, suggesting contextual differences in post-diagnosis behavior modification, risk awareness, and the impact of the health promotion program [27].

Previous research in Viet Nam and other LMICs has typically examined NCD risk factors as single exposures or explored behavioral-metabolic associations as causal effects. Only a few studies have assessed clustering, often in the form of simple counts, without evaluating temporal changes or identifying specific clustering patterns. Collectively, these studies report an increasing burden of NCD risk factors across LMICs [28, 29]. This trend is often attributed to economic development, population aging, and shifts in diet and lifestyle [29, 30], although these factors were not directly examined in this study. Using Asian-calibrated thresholds, this study provides updated national estimates of NCD metabolic risk clustering in Viet Nam and tracks its increase between 2015 and 2021. By examining clustering trends over time, we identified elevated blood glucose as a major contributor to the rising burden. The application of LCA in this study allowed the identification of data-driven behavioral-metabolic profiles without assuming direct linear relationships between individual behavioral and metabolic risk factors [31].

This study used nationally representative STEPS data, which employed standardized protocols across survey rounds, enabling robust comparisons over time. However, behavioral risk factors in STEPS were self-reported and may be subject to recall bias. The repeated cross-sectional design enables temporal comparisons but still limits causal inference. In addition, the latent class analysis and subsequent regression models were estimated without incorporating survey weights or complex survey design variables because Stata lacked methodological support for latent class estimation. Therefore, latent class prevalence estimates and regression parameters should be interpreted with appropriate caution. Although standardized protocols were applied, unobserved differences in measurement conditions (e.g., fasting status or field procedures) may have influenced estimates of metabolic indicators, particularly fasting blood glucose. Furthermore, some contextual factors (e.g., dietary patterns, healthcare access, and urbanization) were not consistently measured across survey rounds and could not be fully accounted for in the analysis.

The rapid rise in fasting glucose and co-occurring metabolic risks highlights the need for earlier detection and integrated management of multiple risks. With NCD services decentralized to commune health stations, Viet Nam should prioritize routine multi-risk screening (blood pressure, BMI/waist, fasting glucose, and, where feasible, cholesterol), simple risk stratification, and coordinated follow-up, with particular attention to high-risk groups such as men with unhealthy behaviors and older adults with metabolic risks.

## Data Availability

Data used in this study are publicly available through the World Health Organization STEPS website: https://extranet.who.int/ncdsmicrodata/index.php/home.
