# Supplementary material for: Rising clustering of metabolic risk factors and behavioral-metabolic profiles in Viet Nam, 2015–2021: repeated cross-sectional surveys
Source: Int J Public Health. 2026 Jun 19;71:1609281. doi: 10.3389/ijph.2026.1609281 (PMC13370801; doi:10.3389/ijph.2026.1609281)
Supplement: Supplementary file 1 [file Table1.docx]

***Supplementary Table 1. Model fit statistics for latent class analysis of behavioral and metabolic risk indicators, WHO STEPwise Approach to NCD Risk Factor Surveillance, Viet Nam, 2021 (2-7 classes)***

| Number of Classes | N | Log-Likelihood (Model) | Degrees of Freedom (df) | AIC | BIC | Entropy |
| --- | --- | --- | --- | --- | --- | --- |
| 2-Class | 3,306 | -16,684.42 | 17 | 33,402.85 | 33,506.61 | 0.52 |
| 3-Class | 3,306 | -16,507.32 | 26 | 33,066.63 | 33,225.32 | 0.50 |
| 4-Class | 3,306 | -16,477.33 | 35 | 33,024.65 | 33,238.27 | 0.51 |
| 5-Class | 3,306 | -16,457.96 | 44 | 33,003.91 | 33,272.47 | 0.55 |
| 6-Class | 3,306 | -16,436.89 | 52 | 32,977.77 | 33,295.15 | 0.48 |
| 7-Class | 3,306 | -16,428.81 | 57 | 32,971.62 | 33,319.52 | 0.56 |
